# From cultural awareness to safety in Canadian speech‑language pathology practice: A scoping review protocol

**DOI:** 10.1371/journal.pone.0347949

**Published:** 2026-08-10

**Authors:** Mariam Komeili, Jessi Robinson, Hannah Akrigg

**Affiliations:** 1 School of Rehabilitation Science, College of Medicine, University of Saskatchewan, Saskatoon, Saskatchewan, Canada; 2 Health Sciences Library, University of Saskatchewan, Saskatoon, Saskatchewan, Canada; 3 Department of Linguistics, University of Saskatchewan, Saskatoon, Saskatchewan, Canada; Father Muller Charitable Institutions, INDIA

## Abstract

Despite growing attention to cultural safety and cultural responsiveness in health professions, no scoping review has focused exclusively on Canadian Speech‑Language Pathology (S-LP) practice across Indigenous peoples and other equity‑deserving groups. Existing reviews often combine S-LP with other rehabilitation professions, focus on either pediatric or adult populations but not both, or limit analysis to a single service‑delivery context. This scoping review protocol outlines an approach to mapping Canadian literature on culturally safe and culturally responsive S-LP practices across populations, contexts, and the lifespan, examining Indigenous peoples as distinct Nations and collective rightsholders rather than grouping them within other equity‑deserving populations. Guided by the PRISMA‑ScR framework, the review will systematically search peer‑reviewed and grey literature describing Canadian S-LP practice. A culturally informed continuum spanning cultural awareness, sensitivity, competence, responsiveness, and safety will guide data charting and synthesis. Terminology such as *trauma‑informed* will be included as a search concept due to its prevalence in the literature and will be interpreted using strength‑based and culturally safe lenses. The review will produce a descriptive map of how culturally safe and responsive practices are defined and implemented in Canadian S-LP literature, including strategies addressing Indigenous peoples and other equity‑deserving groups, and will identify key evidence gaps. Findings will inform S-LP education, training, and practice by clarifying how culturally safe and responsive approaches are currently described and where further research is needed within Canadian contexts.

## Introduction

Speech and language are inseparable from culture; they encode identity, worldview, relational norms, and culturally grounded ways of interacting with the world [1]. Because Speech‑Language Pathologists (S-LPs) work directly with communication, effective practice must engage the cultural, linguistic, and social contexts that shape how people use and value language. Accordingly, cultural competence, responsiveness, and humility are essential for addressing social determinants of health and educational barriers affecting culturally and linguistically diverse (CLD) populations [2,3]. Despite this imperative, S-LP assessment and intervention in Canada have largely been shaped by white, Eurocentric models that do not align with the lived realities or communication systems of many communities [4]. This misalignment is especially pronounced for First Nations, Inuit, and Métis peoples, for whom standardized assessments frequently fail to recognize cultural and linguistic strengths. Such limitations contribute to misidentification and misdiagnosis, with downstream consequences for educational trajectories, psychosocial wellbeing, and health outcomes [4–6].

### Why indigenous peoples have unique research needs

First Nations, Inuit, and Métis peoples are recognized as “Aboriginal peoples” under Section 35(2) of the Constitution Act, 1982, which affirms the constitutional recognition of Aboriginal and treaty rights held collectively by Indigenous Nations and communities. They therefore cannot be conceptualized as one minority group among many; their distinct status as Indigenous Nations and collective rightsholders with specific languages, histories, and legal recognition requires approaches grounded in cultural safety and strength‑based, relational practice. While the term *trauma‑informed* is widely used within Canadian health and rehabilitation literature and is included in this review for methodological completeness, it is applied here with attention to Indigenous‑identified critiques of deficit‑based framing and an emphasis on resilience, continuity, and self‑determination.

Other marginalized groups in Canada, often described as equity‑deserving, such as racialized communities, immigrants and refugees, and 2SLGBTQIA+ individuals, also experience cultural, linguistic, and systemic barriers to equitable S-LP services [2,3]. However, their sociopolitical contexts differ markedly from those of Indigenous Nations. Accordingly, this review examines Indigenous peoples and other populations experiencing inequities in parallel but never interchangeably, maintaining a distinctions‑based approach throughout.

For clarity and consistency, the term equity‑deserving groups will be used throughout this protocol to refer to marginalized, non‑Indigenous populations experiencing structural and systemic inequities. This protocol and the resulting scoping review are guided by a strength‑based framework that seeks to foreground assets, resilience, and culturally grounded practices. At the same time, recognizing methodological requirements for comprehensive literature retrieval and synthesis, the review will engage with a range of terminology used within the existing literature. Such terminology will be critically examined and interpreted through culturally safe, relational, and strength‑based lenses.

### Problem statement and rationale

Research examining equity and power in S-LP has shown that historical and social factors continue to shape research, education, and professional norms. In particular, such work has drawn attention to how colonialism, racism, cisnormativity, ableism, and deficit‑based medical models have influenced research priorities, professional training, and clinical practice, shaping what is assessed, how communication differences are interpreted, and which language practices are positioned as typical or disordered. A 2023 scoping review by [7] synthesized 39 articles examining such structures of harm within communication‑related disciplines and identified colonialism as the most frequently discussed influence, with Indigenous peoples most often represented across critical analyses. Importantly, the authors noted that this body of work remains **largely conceptual rather than practice‑based**, with limited examination of how S-LP services are delivered in real‑world Canadian contexts. Specifically, existing scholarship does not adequately address how cultural safety is implemented in S-LP practice, how culturally responsive approaches are enacted within clinical encounters, or how commonly used frameworks such as those described as trauma‑informed are operationalized in Canadian S-LP service delivery. This gap directly justifies the present scoping review protocol, which shifts the focus from conceptual critique to **mapping applied, practice‑oriented evidence in S-LP services and practices.**

Across rehabilitation professions and within S-LP specifically evidence describing how clinicians enact cultural awareness, sensitivity, competence, humility, responsiveness, and safety in Canadian contexts remains fragmented. Existing reviews identify persistent barriers to culturally responsive S-LP practice, including language mismatch, Western‑centric service delivery models, limited availability of culturally relevant assessment tools, and insufficient clinician training. At the same time, promising facilitators have been identified, such as flexible delivery approaches, culturally meaningful materials, and transparent explanations of health systems; however, implementation remains inconsistent [8]. In contrast, intervention adaptation studies demonstrate that culturally and linguistically responsive S-LP practices are associated with improved engagement and outcomes [1] as well as enhanced social validity through better alignment with family priorities [9].

Clarifying **who is encompassed within culturally and linguistically diverse (CLD) populations in S-LP research and practice** is therefore critical. [10] recommend defining CLD populations as individuals born in non–English‑speaking countries and/or those who do not speak English at home, while emphasizing that constitutionally recognized Indigenous peoples must be considered separately due to their distinct cultural, historical, and political contexts. These distinctions underscore the need for a scoping review focused specifically on **Canadian S-LP practice**, examined through a culturally safe and distinctions‑based lens.

### What prior reviews have not addressed

Despite several relevant reviews, **no prior scoping review has focused exclusively on Canadian S-LP practices** to examine how cultural responsiveness, cultural safety, and approaches described in the literature as trauma‑informed are enacted within clinical encounters. Existing syntheses of cultural competence and S-LP practice have typically emphasized either pediatric or adult populations – but not both together – or have examined a single service‑delivery context in isolation. This protocol addresses that gap.

Many scoping reviews have further grouped S-LP alongside other rehabilitation professions (e.g., occupational therapy, physiotherapy), rather than examining S-LP practice as a distinct discipline. Reviews that do focus on S-LP adaptation frequently emphasize early intervention and do not include adult or lifespan‑wide practices. At the same time, research in S-LP has highlighted how structural inequities and historically rooted assumptions influence how communication differences are defined, assessed, and addressed in practice. However, this work has largely focused on conceptual critique rather than examining how culturally responsive and culturally safe approaches are implemented within everyday Canadian S-LP service delivery. Similarly, broader rehabilitation reviews that include S-LP rarely evaluate the implementation of cultural safety, critically examine trauma‑informed frameworks, or attend to Indigenous‑specific and distinctions‑based approaches.

Accordingly, this scoping review protocol addresses a significant gap by **mapping applied evidence** on culturally safe and culturally responsive S-LP practices in Canada. The review will examine how such practices are described and implemented for Indigenous peoples and other equity‑deserving groups examined in parallel but not interchangeably, and across multiple service‑delivery models, including health care, schools, private practice, and both assessment and intervention contexts.

### Conceptual framework: The cultural continuum

This review is guided by the **cultural continuum** developed by [11]. In keeping with Section 35(2), “Aboriginal” refers collectively to First Nations, Inuit, and Métis peoples, examined here as a distinct rights holding population, while other equity deserving groups (racialized communities, immigrants, refugees, 2SLGBTQIA+) are considered separately given their different sociopolitical positions and experiences of inequity. The continuum includes: **cultural awareness** (recognizing and accepting cultural differences; foundational but insufficient on its own); **cultural sensitivity** (respecting differences and acknowledging variability within and across groups); **cultural competence** (developing the knowledge, skills, and attitudes to adapt care to diverse cultural and linguistic needs); **cultural humility** (lifelong self-reflection and attention to power imbalances in equitable partnerships); **cultural responsiveness** (adapting practice to ensure cultural relevance, meaning, and respect); and **cultural safety** (addressing systemic inequities, colonial structures, and power imbalances to create client defined safety).

### Purpose and objectives

**The purpose** of this scoping review will be to map the Canadian literature on S-LP practices across the cultural continuum-from awareness to safety to identify strategies used to address cultural and linguistic needs and highlight knowledge gaps. **Objectives** include informing future research, guiding clinical practice, and supporting policy development aimed at reducing inequities and promoting culturally safe communication care for Indigenous peoples and other equity deserving communities in ways that honour their distinct cultural, historical, and sociopolitical contexts.

## Research questions

1) How culturally responsive are Speech Language Pathology (S-LP) services for Indigenous peoples (First Nations, Inuit, and Métis) and other equity‑deserving groups in Canada, and what similarities or differences are reported?
2) How are culturally safe and culturally responsive practices implemented across Canadian Speech‑Language Pathology (S-LP) contexts, and what strategies specifically address Indigenous cultural needs in comparison to other equity‑deserving groups?
3) What evidence exists regarding progress toward cultural competence and safety in Speech‑Language Pathology (S-LP) practice for Indigenous peoples and other equity‑deserving groups in Canada, and what gaps remain?

## Methods

This scoping review will use the methodological frameworks outlined by [12] and later expanded by [13] to ensure the project adheres to a high standard of methodological rigor and transparency throughout the processes of study identification, selection, data charting, and analysis. In accordance with current best practices, this review will also adhere to the Preferred Reporting Items for Systematic Reviews and Meta-Analyses extension for scoping reviews (PRISMA-ScR) [14]. The PRISM-ScR checklist is provided in S2 Appendix. The review question and eligibility criteria were guided by the Population–Concept–Context (PCC) framework as shown in Table 1 below.

**Table 1:** Population-Concept-Context (PCC) framework.

Component	Description
**Population**	Indigenous Peoples (First Nations, Inuit, Métis) and other equity-deserving populations, including racialized groups, immigrants and refugees, 2SLGBTQIA+ individuals, and other marginalized communities experiencing structural or systemic inequities in Canada.
**Concept**	Culturally responsive, culturally safe, trauma-informed, and equity-oriented practices, frameworks, assessments, and interventions in speech-language pathology. This includes concepts across the cultural continuum such as cultural awareness, sensitivity, competence, humility, responsiveness, and safety.
**Context**	Speech-language pathology practice in Canada, including services delivered across healthcare, educational, community, and private practice settings, across the lifespan.

This scoping review protocol is registered with OSF Registries Jan 28, 2026: https://doi.org/10.17605/OSF.IO/735QB.

### Eligibility criteria

#### Population.

Broad population inclusion criteria were intentionally selected to align with the objectives of a scoping review, which aims to map the scope, nature, and characteristics of existing literature rather than evaluate intervention effectiveness. This inclusion criteria allows for comparative examination of how culturally responsive and equity-oriented practices are conceptualized and implemented across diverse sociocultural and historical contexts.

Sources will be considered eligible if they focus on, include, or are explicitly relevant to populations that experience social, cultural, or structural marginalization, including but not limited to:

- Indigenous peoples, including First Nations, Inuit, and Métis in Canada.
- Racialized communities, as defined by authors using terminology such as racialized, ethnic minority, or similar descriptors reflecting experiences of systemic racism.
- Immigrants and refugees, including recent and longstanding immigrant populations.
- 2SLGBTQIA+ (Two-Spirit, Lesbian, Gay, Bisexual, Transgender, Queer or Questioning, Intersex, and Asexual peoples) including diverse sexual orientations and gender identities.
- Other marginalized or equity deserving communities, where marginalization is described in relation to social, cultural, linguistic, structural, or historical inequities relevant to healthcare, education, or communication related services.

#### Concept.

Studies that examine, describe, or discuss culturally responsive, culturally safe, trauma‑informed, or equity‑oriented practices, frameworks, assessments, or interventions relevant to S-LP or closely related communication‑focused disciplines (e.g., Communication Sciences and Disorders). These concepts related to culturally responsive or culturally safe practices are extremely varied in terminology as it is still an emerging area of professional practice. To help guide this project’s scope, we will be using the definitions outlined in [11] to ensure identification of relevant material with regard to this concept.

#### Context.

Eligible sources will include studies or resources conducted within clinical, educational, community, or research settings. Only sources based in Canada will be included to ensure relevance to the Canadian sociocultural context.

### Types of sources

The following types of sources will be included: empirical studies (quantitative, qualitative, or mixed methods), reviews, conceptual papers, and relevant grey literature (including dissertations and theses, community organization reports, government documents and professional organization policies) that contribute to understanding practices with the populations of interest.

### Exclusion criteria

Resources will be excluded if:

- Focused on a population exclusively outside of Canada.
- Focus exclusively on populations not experiencing social or structural marginalization relevant to the review objectives.
- Does not address equity-oriented, or cultural responsiveness related concepts.
- Are unrelated to speech language pathology, communication, rehabilitation or health related practices.
- Consist solely of opinion pieces or commentary without substantive discussion relevant to the review aims.
- Not published between 2000–2026, articles published outside these dates will not be included.
- Study not written in English: only studies published in English will be included due to limitations in the research team’s ability to reliably interpret and analyze full-text publications in other languages. The exclusion of non-English studies is acknowledged as a limitation.

### Handling of ambiguous population information

If the population is unclear during title and abstract screening, studies will be retained for full-text review. If the identity of the population or marginalization status remains ambiguous at the full-text stage, eligibility will be determined through discussion among reviewers, with reference to the review objectives and whether the study meaningfully engages with issues of cultural responsiveness, equity, or marginalization.

### Ensuring shared interpretation of eligibility criteria

Prior to formal screening, all reviewers will pilot the eligibility criteria on a sample of studies to ensure a shared understanding of inclusion and exclusion criteria. Team discussions will be used to clarify interpretations and refine criteria as needed. Any refinements will be documented to ensure transparency and consistency in the screening process.

### Search strategy

Our search strategy will aim to locate both published studies and grey literature resources. With the support of a health sciences librarian (JR), we developed an initial search in MEDLINE (Ovid) using a combination of keywords and Medical Subject Headings (MeSH). Seed articles identified during early stages of the project, along with the subject‑matter expertise of the research team, informed the development of a comprehensive search strategy. This strategy was iteratively refined to incorporate emerging terminology relevant to the research question.

The search process culminated in a final strategy that used keywords appearing in the title, abstract, or author‑supplied keywords fields, in combination with database‑specific controlled vocabulary (e.g., MeSH terms). Due to the breadth and variability of terminology associated with the concept of cultural safety, it was determined that these terms would be excluded from the search string and instead addressed during the screening stage of the review. The complete MEDLINE (Ovid) search strategy is provided in S1 Appendix. The databases to be searched include MEDLINE, Embase, CINAHL, Web of Science, and Academic Search Complete.

A structured grey literature search will be conducted to capture relevant non-indexed sources. Sources will include organizational and policy documents, professional guidelines, dissertations and theses, and reports relevant to speech-language pathology and culturally responsive practice in Canada. Specifically, grey literature will be identified through targeted searches of:

- Professional and regulatory organizations (e.g., Speech-Language & Audiology Canada, provincial regulatory bodies)
- Government and health authority websites (federal and provincial)
- Community and non-profit organizations relevant to equity-deserving populations
- Institutional repositories and academic databases (e.g., ProQuest Dissertations and Theses)
- Grey literature databases (e.g., Canada Commons)
- Targeted Google searches (screening the first 100 results per search)

Search procedures will involve combinations of keywords related to speech-language pathology (e.g., “speech-language pathology,” “speech therapy,” “communication disorders”), cultural responsiveness (e.g., “cultural safety,” “cultural competence,” “cultural responsiveness,” “trauma-informed”), and population terms (e.g., “Indigenous,” “First Nations,” “Inuit,” “Métis,” “immigrant,” “refugee,” “equity-deserving”). Search strings will be adapted for each source, and screening will follow the Population–Concept–Context (PCC) framework used for peer-reviewed literature.

Grey literature sources will be eligible for inclusion if they meet the same inclusion criteria as peer-reviewed studies and provide substantive discussion of culturally responsive or culturally safe practices in S-LP within Canadian contexts. Sources will be excluded if they do not address the concept of interest, are not relevant to S-LP or lack sufficient detail to support data extraction.

All grey literature searches will be documented to ensure transparency and reproducibility, including search terms used, dates of search, sources consulted, and number of records identified and retained. These results will be reported in the PRISMA flow diagram and described in the methods and results sections.

### Study selection and screening

Eligibility criteria will be applied consistently throughout both title/abstract and full-text screening stages. Studies will be included if they meet all the following criteria:

1. Population/focus: this study examines minority communities in Canada, these include:
  - Visible minorities: Racialized groups identified by race, colour or ethnic origin.
  - Invisible minorities: Religious minorities, linguistic minorities, and LGBTQ2S+ identifying individuals.
  - Studies involving families, caregivers, or service providers will be included when findings relate to minority experiences within S-LP.
2. Concept: The article directly addresses culturally safe, responsive or inclusive S-LP practices, this includes assessment, intervention and service delivery.
3. Context: Research is conducted in Canada, or findings can be meaningfully applied to the Canadian S-LP context, this includes healthcare, community, educational and rehabilitation settings.
4. Evidence Types: Peer reviewed empirical studies, reviews, grey literature and conceptual/ theoretical papers relevant to cultural safety in S-LP
5. Language: Studies written in English
6. Date Range: Published between 2000–2026

The systematic review platform Covidence [Covidence systematic review software, Veritas Health Innovation, Melbourne, Australia] will be used for citation management, blinded screening, conflict resolution, and documentation to support PRISMA-ScR reporting.

Before the formal abstract/title screen, the two primary reviewers will complete two pilot rounds to ensure consistent interpretation of the eligibility criteria. Each round will include a number of randomly selected citations, this number being dependent on the total number of articles found. Both reviewers will independently screen selected citations, meeting to discuss discrepancies, refine definitions and adjust screening rules after each round as necessary. A minimum 95% agreement threshold is required to pass each pilot round. Pilot rounds will be repeated until this threshold is reached. Following the two successful pilot screens the two independent reviewers will screen all titles and abstracts using the eligibility criteria. Records will be coded as “include”, “exclude” or “uncertain”. If information in the abstract/title is insufficient to judge eligibility, the record will automatically be advanced to full-text review. If disagreements arise, the reviewers will first attempt to resolve the disagreement through discussion, if a consensus cannot be achieved a third reviewer will serve as a tie braker. The review software will track decisions, conflicts and screening outputs for PRISMA documentation.

Before the official full-text screening, the two reviewers will screen a random sample of full-text articles provided by the review software, independently applying the full eligibility criteria. Reviewers will compare decisions, discuss any challenges, including but not limited to ambiguous methodologies, unclear populations, or culturally safe practice terminology. A 95% agreement threshold is required to proceed to full screening. Any revisions to the screening guide will be documented. After successfully passing the pilot round, each full-text article will be independently screen by two reviewers, being coded as “include”, “exclude”, or “needs clarification”, unresolved disagreements will be adjudicated by a third reviewer. When full texts contain unclear or missing information, reviewers will attempt to retrieve supplementary files or companion reports, if the uncertainty remains and the study meets eligibility criteria the team will err toward inclusion to avoid premature exclusion. Screening will be managed using the review software.

### Data management and availability

This scoping review will generate data related to the screening process (e.g., inclusion and exclusion decisions), extracted study characteristics, and findings. These data will be managed using Covidence systematic review software and saved CSV file type for data extraction, coding, and version control.

All data records, including screening decisions and extracted data, will be stored on secure, password-protected institutional systems in accordance with University of Saskatchewan data management policies. In line with PLOS ONE data-sharing requirements, no original data are generated for this protocol. Upon completion of the review, relevant data (e.g., extracted datasets and summary tables) will be made available in a public repository (e.g., osf repository, HARVEST USask repository), or provided upon request. This approach supports transparency, reproducibility, and compliance with PLOS ONE data-sharing policies.

### Data charting/data extraction

The initial data charting form will be developed based on the research questions, eligibility criteria, and conceptual frameworks relating to cultural safety, minority populations and S-LP contexts. It will include fields relating to citation details, study design, participants, setting, cultural safety components, interventions and outcomes relevant to the review. The form will be reviewed by content experts, an information specialist, external advisors. A potential structured meeting will follow, during which the team will extract data from one example article to identify unclear fields or missing elements. Data charting will be iterative, and all revisions will be documented.

Data items to be collected include:

1. Citation details: authors, year title, source, DOI/URL, funding, conflicts of interest,
2. Study characteristics: region, S-LP setting (school, healthcare, community, private, rehabilitation), design, aims.
3. S-LP domain: speech, language, fluency, voice, swallowing, AAC, hearing, etc.
4. Service stage: assessment, intervention, follow-up, care coordination.
5. Cultural safety components: strategies, frameworks, adaptations.
6. Outcomes and findings: client/family outcomes, function and equity outcomes, satisfaction.
7. Notes: limitations, research gaps, contextual features

Data extraction rules, coding definitions, decision trees and worked examples will be stored in a secure, shared cloud-based workspace, with version history tracking reviewer initials, timestamps and rationales for all updates.

Before official extraction two pilot extraction rounds will be completed. A small sample of included studies will be selected by the software, both reviewers will then independently extract these studies, meeting afterwards to compare results and refine definitions as needed. A 95% agreement threshold is required to pass each pilot round. Following two successful pilot rounds main data extraction will begin. One reviewer will extract data, and a second will verify all fields. Discrepancies will be resolved through discussion, unresolved items will be adjudicated by a third reviewer. Missing or unclear information will be coded as “not reported”, “unclear” or “requires clarification”, reviewers will check supplementary materials and companion reports. If critical information remains missing, the team will contact the listed first author up to three times over 21 days. At this stage studies will not be excluded based solely on missing information. Data will be extracted using the software management system or Excel, with full audit trails maintained.

### Stakeholder consultation

Stakeholder consultation will be undertaken to support the culturally appropriate and respectful representation of findings, rather than to inform study selection, data extraction, or interpretation of results. This reflects the study’s focus on how culturally responsive and culturally safe practices are described in the literature, while recognizing the potential impact of how findings are written. Where feasible, stakeholders will include community partners and knowledge users connected to Indigenous communities, newcomer and immigrant populations, and/or other equity-deserving groups. The purpose of this consultation is to ensure that the language, framing, and representation of findings are culturally appropriate and aligned with principles of cultural safety.

Consultation will occur primarily during write-up stage when findings are being presented to ensure authors are using culturally responsive and strength based terminology. Stakeholders may be invited to provide input on language use and framing of findings. Feedback will inform revisions to language and presentation but will not influence analytic conclusions. Stakeholder input will be documented in a manner that respects participant preferences, including options for named, collective, or no acknowledgment. The extent of consultation will be reported transparently, and any limitations will be acknowledged.

### Synthesis and presentation of results

After data extraction is complete, the research team will conduct a systematic data-cleaning process that includes checking for missing data and marking items as “not reported”, “unclear”, or “not applicable”, standardizing terminology, corrected inconsistencies across extraction fields, removing duplicate information, ensuring consistency between the final dataset and predetermined data items to maintain internal consistency. All changes during data cleaning will be documented in a shared audit log.

Data cleaning and synthesis will be managed using a combination of Excel for data cleaning, coding and version control and the software management system for theme identification and coding or qualitative findings and for screening history and record keeping. These tools support transparent tracking, collaborative editing and a clear thematic analysis.

A PRISMA flow diagram (PRISMA-ScR format) will be used to document the number or records identified, number screened, and number included/excluded at each stage of the process. This diagram will provide a clear visual summary of all aspects of the study selection process.

The synthesis of the data will follow an iterative, narrative and descriptive approach, this will include a descriptive numerical summary which will be a quantitative overview on the number of included studies, study locations, types of minority groups represented, study design and settings, and types of S-LP interventions and cultural-safety strategies studied. Qualitative findings will be synthesized using a coding framework developed from extracted data, on the software management system, cultural safety literature, stakeholder or community partner input when possible (e.g., S-LP practitioners, minority group representatives) these themes may include but are not limited to barriers to culturally safe S-LP services, promising practices, structural considerations, lived experiences of minority communities, gaps in research and service. Stakeholder contributions will help ensure that the synthesis reflects the real-world applications of the review and the needs of the community.

Results will be presented in multiple formats, including tables representing study characteristics, minority groups represented, cultural safety strategies used, outcomes and key findings, and gaps and recommendations. Figures and diagrams will be used such as the PRISMA flow diagram, Bar or Pie Charts describing frequencies such as study types and populations and conceptual maps that demonstrate cultural safety components in S-LP may be used. A narrative synthesis may be used to integrate key themes, implications for practice and policy, as well as any implications for future research.

Each data item will have a specific use in data synthesis. Citation details will be used in a basic descriptive table to map publication trends. Study design will be used to group studies by methodology to identify gaps. Population characteristics will be used to categorize visible vs invisible minority representation to assess the inclusivity of the study. Setting and context will be used to compare cultural safety practices across S-LP environments, similarly intervention and practice will be used to identify culturally safe strategies and adaptations. Cultural safety components will be used in thematic analysis to help develop a model of what works. Outcomes and findings will be used to synthesize the reported impacts of the studies and to identify what strategies work. Barriers and facilitators will be used in thematic coding to help inform practice recommendations, as gaps and future directions will be used to inform implications for research and policy.

## Supporting information

S1 Appendix MEDLINE (Ovid) search strategy. (DOCX)

S2 Appendix PRISMA-ScR checklist. (DOCX)
