# Supplementary material for: From cultural awareness to safety in Canadian speech‑language pathology practice: A scoping review protocol
Source: PLoS One. 2026 Aug 10;21(8):e0347949. doi: 10.1371/journal.pone.0347949 (PMC13456271; doi:10.1371/journal.pone.0347949)
Supplement: Supplementary file 1 [file pone.0347949.s001.docx]

Medline Search String Example Ovid MEDLINE(R) ALL <1946 to January 27, 2026>

1 (Inuit or Metis or Indigenous or Inuk or "First Nations" or Aboriginal or "visible miniorit*" or LGBT* or gay or transgender or "two spirit" or Queer or Afro-Canadian or Black or BIPOC or refugee* or immigrant* or racialized or "Asylum seeker*" or newcomer or Chinese or Black or Filipin* or Arab* or Latin* or Asian or Korean or Japanese or Bangladeshi or Indian* or Pakistani or Punjabi or "Sri Lankan*" or Nigerian* or African* or Sudanese or Ghanian* or Syrian* or Farsi or Persian* or Iranian* or Vietnamese or Cambodian* or Laotian* or Thai or "Middle Eastern" or Afghan* or "Bi-racial" or "Multi-racial" or "mixed race" or Haitian* or Congolese or Nigerian*).ab,kw,ti. 1701428

2 exp "health disparate minority and vulnerable populations"/ or exp "sexual and gender minorities"/ or exp Minority Groups/ or exp Indigenous Peoples/ or Health Services, Indigenous/ 212483

3 1 or 2 1784043

4 ("Speech Disorder*" or "Speech Delay*" or "Speech impair*" or "Language disorder*" or "Language delay*" or "Language Impair*" or "Communication disorder*" or "Communication delay*" or "Communication Impair*" or SLP).ab,kw,ti. 24844

5 (Speech adj2 (patholog* or language or therap*)).ab,kw,ti. 21367

6 "rehabilitation of speech and language disorders"/ or language therapy/ or myofunctional therapy/ or speech, alaryngeal/ or speech therapy/ or voice training/ or communication disorders/ or language disorders/ or learning disabilities/ or Speech-Language Pathology/ 38938

7 4 or 5 or 6 69459

8 (Canad* or "British Columbia" or "Colombie Britannique" or Alberta* or Saskatchewan or Manitoba* or Ontario or Quebec or "Nouveau Brunswick" or "New Brunswick" or "Nova Scotia" or "Nouvelle Ecosse" or "Prince Edward Island" or Newfoundland or Labrador or Nunavut or NWT or "Northwest Territories" or Yukon or Nunavik or Inuvialuit).ab,kw,ti. 234995

9 (Abbotsford or Airdrie or Ajax or Aurora or Barrie or Belleville or Blainville or Brampton or Brantford or Brossard or Burlington or Burnaby or Caledon or Calgary or Cape Breton or Chatham Kent or Chilliwack or Clarington or Coquitlam or Drummondville or Edmonton or Fredericton or Fort McMurray or Gatineau or Granby or Grande Prairie or Sudbury or Guelph or Halton Hills or Iqaluit or Inuvik or Kamloops or Kawartha Lakes or Kelowna or Kingston or Kitchener or Langley or Laval or Lethbridge or Levis or Longueuil or Maple Ridge or Markham or Medicine Hat or Milton or Mirabel or Mississauga or Moncton or Montreal or Nanaimo or New Westminster or Newmarket or Niagara Falls or Norfolk County or North Bay or North Vancouver or Oakville or Oshawa or Ottawa or Peterborough or Pickering or Port Coquitlam or Prince George or Quebec City or Red Deer or Regina or Repentigny or Richmond or Richmond Hill or Saanich or Saguenay or Saint John or Saint-Hyacinthe or Saint-Jean-sur-Richelieu or Saint-Jerome or Sarnia or Saskatoon or Sault Ste Marie or Sherbrooke or St Albert or St Catharines or St John's or Strathcona County or Surrey or Terrebonne or Thunder Bay or Toronto or Trois-Rivieres or Vancouver or Vaughan or ((Cambridge or (Halifax or Hamilton or London or Victoria or Waterloo or Welland or Whitby or Windsor)) not (UK or Britain or United Kingdom or England or Australia)) or Whitehorse or Winnipeg or Wood Buffalo or Yellowknife).ti,ab,kw. 163035

10 exp Canada/ 197031

11 8 or 9 or 10 435592

12 3 and 7 and 11 80
