# Supplementary material for: From cultural awareness to safety in Canadian speech‑language pathology practice: A scoping review protocol
Source: PLoS One. 2026 Aug 10;21(8):e0347949. doi: 10.1371/journal.pone.0347949 (PMC13456271; doi:10.1371/journal.pone.0347949)
Supplement: Supplementary file 3 [file pone.0347949.s003.docx]

**Response to Reviewer and Editor**

We would like to thank the Academic Editor and Reviewers for their time and thoughtful feedback on our manuscript. We appreciate the constructive comments and suggestions provided, which have helped strengthen the clarity, rigor, and transparency of the protocol.

We have carefully reviewed all comments and have revised the manuscript accordingly. All changes have been incorporated into the revised manuscript and are detailed below.

**1. Population–Concept–Context (PCC) Framework**

The protocol would benefit from a clearer presentation of the Population, Concept, and Context (PCC) framework that underpins the review question. While these elements can be inferred from the text, explicitly presenting them in a dedicated table would improve transparency, methodological rigor, and alignment with established scoping review guidance. The PCC framework should clearly identify the target population, the concepts being examined, and the contextual boundaries of the review.

***Response:*** *The table below has been added to the manuscript and labelled as Table 1*

| ***Component*** | ***Description*** |
| --- | --- |
| ***Population*** | *Indigenous Peoples (First Nations, Inuit, Métis) and other equity‑deserving populations, including racialized groups, immigrants and refugees, 2SLGBTQIA+ individuals, and other marginalized communities experiencing structural or systemic inequities in Canada.* |
| ***Concept*** | *Culturally responsive, culturally safe, trauma‑informed, and equity‑oriented practices, frameworks, assessments, and interventions in speech‑language pathology. This includes concepts across the cultural continuum such as cultural awareness, sensitivity, competence, humility, responsiveness, and safety.* |
| ***Context*** | *Speech‑language pathology practice in Canada, including services delivered across healthcare, educational, community, and private practice settings, across the lifespan.* |

**2. Stakeholder Consultation**

The manuscript indicates stakeholder involvement; however, important details regarding this process are currently lacking. The authors should clearly specify:

***Response:*** *Thank you for this helpful suggestion. We have revised the manuscript to explicitly address each component of stakeholder consultation as outlined below:*

- ***Who the stakeholders are:*** *We now specify that stakeholders may include community partners and knowledge users such as community members, Elders, and individuals connected to Indigenous communities, newcomer and immigrant populations, and other equity‑deserving groups.*
- ***Rationale for selecting these stakeholders:*** *The rationale is to support culturally appropriate and respectful representation of findings, ensuring that the language, framing, and presentation of results align with principles of cultural safety and do not misrepresent the communities discussed.*
- ***Stage(s) of consultation:*** *Consultation will occur during the* ***write‑up stages*** *of the review.*
- ***Objectives of stakeholder consultation:*** *The objectives are to support culturally safe language use, ensure appropriate representation of communities, and identify any potentially unclear or inappropriate framing in how findings are presented.*
- ***Documentation and incorporation of feedback:*** *Stakeholder input will be documented in a manner that respects participant preferences. Feedback will inform revisions to* ***language and presentation*** *of findings but will not influence study selection, data extraction, or interpretation of results. Contributions will be acknowledged based on participant consent (named, collective acknowledgment, or not acknowledged).*

*We have also clarified that stakeholder consultation may not always be feasible, and the extent of consultation will be reported transparently with limitations noted in the manuscript*.

**3. Grey Literature Search Strategy**

The protocol currently provides insufficient detail regarding the planned grey literature search.

***Response:*** *Thank you for this valuable suggestion. We have revised the manuscript to expand the grey literature search strategy and explicitly address each component as outlined below:*

- ***Sources to be searched:*** *We now specify that grey literature will be identified through targeted searches of professional and regulatory organizations, government and health authority websites, community and non‑profit organizations, institutional repositories (e.g., ProQuest Dissertations and Theses), grey literature databases (e.g., Canada Commons), and Google searches.*
- ***Search procedures and search terms:*** *We clarify that combinations of keywords related to Speech‑Language Pathology, cultural responsiveness, and populations of interest will be used, and that search strings will be adapted for each source.*
- ***Inclusion and exclusion criteria for grey literature:*** *We specify that grey literature sources must meet the same Population–Concept–Context criteria as peer‑reviewed literature and provide substantive discussion of culturally responsive or culturally safe practices in Canadian S-LP practices and/or services.*
- ***Documentation and reporting methods:*** *We state that all grey literature searches will be documented, including search terms, dates, sources consulted, and records identified, and that these will be reported in the PRISMA flow diagram and described in the manuscript.*

*These revisions have been incorporated to improve transparency, rigor, and reproducibility.*

**4. Language Restrictions**

The review focuses on the Canadian context, where both English and French are official languages. The authors should either:

* Expand the search strategy to include French-language publications, or
* Provide a clear methodological justification for restricting the review to English-language sources and discuss the potential implications of this decision.

Failure to address this issue may result in the omission of relevant evidence.

***Response:*** *Thank you for this important point. We have clarified our approach to language inclusion in the Methods section. Due to limitations in the research team’s ability to accurately interpret and analyze full-text French-language publications, the review has been restricted to English-language sources to ensure consistency and rigor in data extraction and analysis. We acknowledge that this decision may result in the exclusion of some relevant evidence, and we now explicitly identify this as a limitation of the review. To enhance transparency, relevant French-language records with available English abstracts, identified during screening will be documented and discussed in the final manuscript.*

**5. Publication Date Range**

There appears to be inconsistency in the stated date limits of the search strategy. Different sections of the manuscript refer to different publication periods (2000–2025 and 2000–2026). The authors should ensure consistency throughout the manuscript and clearly justify the selected timeframe.

***Response:*** *Thank you for highlighting this inconsistency. We have revised the manuscript to ensure that the publication date range is reported consistently throughout. The selected timeframe (2000–2026) is now clearly and consistently specified across all sections of the manuscript.*
6. **Data Management and Availability**

As this is a protocol paper, the manuscript should provide greater clarity regarding management and sharing of the data generated during the review process.

***Response:*** *Thank you for this important suggestion. We have added a dedicated* **Data Management and Availability** *section to the Methods to improve transparency and reproducibility. This section addresses each of the requested elements as follows:*

- ***What review data will be generated:*** *We specify that the review will generate data related to screening decisions (e.g., inclusion and exclusion records), extracted study characteristics, and synthesized outputs.*
- ***Where data will be stored:*** *We clarify that data will be managed using Covidence systematic review software and Microsoft Excel, and stored on secure, password-protected institutional systems.*
- ***Public availability of materials:*** *We state that relevant review data (e.g., extracted datasets and summary tables) will be made publicly available upon completion of the review where feasible, or provided upon reasonable request.*
- ***Compliance with PLOS ONE data-sharing requirements:*** *We note that no original data are generated at the protocol stage and confirm that the study complies with PLOS ONE data-sharing policies.*

*These revisions have been incorporated to enhance clarity, transparency, and reproducibility.*

Overall, the study addresses an important topic and the proposed review is feasible and potentially valuable. However, additional methodological detail and clarification are necessary to ensure transparency, reproducibility, and adherence to current scoping review standards.
