# Supplementary material for: From cultural awareness to safety in Canadian speech‑language pathology practice: A scoping review protocol
Source: PLoS One. 2026 Aug 10;21(8):e0347949. doi: 10.1371/journal.pone.0347949 (PMC13456271; doi:10.1371/journal.pone.0347949)
Supplement: Peer review history

# Peer review: Decision Letter 0

**DOI:** 10.1371/journal.pone.0347949.r001

**Authors:** Ramandeep Kaur

11 Jun 2026

PONE-D-26-17409

From Cultural Awareness to Safety in Canadian Speech‑Language Pathology Practice: A Scoping Review Protocol

PLOS One

Dear Dr. Komeili,

Thank you for submitting your manuscript to PLOS ONE. After careful consideration, we feel that it has merit but does not fully meet PLOS ONE’s publication criteria as it currently stands. Therefore, we invite you to submit a revised version of the manuscript that addresses the points raised during the review process.

==============================

The manuscript addresses an important and timely topic within speech-language pathology and proposes a potentially valuable scoping review examining cultural awareness and cultural safety in the Canadian context. The overall rationale for the review is clear, and the proposed methodology appears feasible. However, several aspects of the protocol require further clarification before the manuscript can meet the standards of transparency and reproducibility expected of a published protocol.

Prior to acceptance, the authors should strengthen the methodological reporting by explicitly presenting the Population–Concept–Context (PCC) framework underpinning the review question. Additional detail is also needed regarding stakeholder consultation, including who will be involved, the purpose of their involvement, when consultation will occur, and how stakeholder input will be incorporated into the review process and findings.

The grey literature search strategy requires more comprehensive description to ensure reproducibility. The authors should clearly specify the sources to be searched, search procedures, eligibility criteria, and methods for documenting and reporting grey literature records. Furthermore, given the Canadian context of the review, the decision to restrict the search to English-language publications should be justified, or the search strategy should be expanded to include French-language literature.

The manuscript should also resolve inconsistencies regarding the stated publication date range and provide a clearer statement regarding management, storage, and sharing of data generated during the review process in accordance with PLOS ONE requirements.

These concerns relate primarily to methodological transparency and reporting rather than the overall study design. Addressing them will substantially strengthen the protocol and improve its reproducibility. I therefore recommend Major Revision.

==============================

Please submit your revised manuscript by Jul 26 2026 11:59PM. If you will need more time than this to complete your revisions, please reply to this message or contact the journal office at plosone@plos.org. When you're ready to submit your revision, log on to https://www.editorialmanager.com/pone/ and select the 'Submissions Needing Revision' folder to locate your manuscript file.

Please include the following items when submitting your revised manuscript:

- A letter that responds to each point raised by the academic editor and reviewer(s). You should upload this letter as a separate file labeled 'Response to Reviewers'.
- A marked-up copy of your manuscript that highlights changes made to the original version. You should upload this as a separate file labeled 'Revised Manuscript with Track Changes'.
- An unmarked version of your revised paper without tracked changes. You should upload this as a separate file labeled 'Manuscript'.

If you would like to make changes to your financial disclosure, please include your updated statement in your cover letter. Guidelines for resubmitting your figure files are available below the reviewer comments at the end of this letter.

If applicable, we recommend that you deposit your laboratory protocols in protocols.io to enhance the reproducibility of your results. Protocols.io assigns your protocol its own identifier (DOI) so that it can be cited independently in the future. For instructions see: https://journals.plos.org/plosone/s/submission-guidelines#loc-laboratory-protocols. Additionally, PLOS ONE offers an option for publishing peer-reviewed Lab Protocol articles, which describe protocols hosted on protocols.io. Read more information on sharing protocols at https://plos.org/protocols?utm_medium=editorial-email&utm_source=authorletters&utm_campaign=protocols.

As the corresponding author, your ORCID iD is verified in the submission system and will appear in the published article. PLOS supports the use of ORCID, and we encourage all coauthors to register for an ORCID iD and use it as well. Please encourage your coauthors to verify their ORCID iD within the submission system before final acceptance, as unverified ORCID iDs will not appear in the published article. *Only*  the individual author can complete the verification step; PLOS staff *cannot*  verify ORCID iDs on behalf of authors.

We look forward to receiving your revised manuscript.

Kind regards,

Ramandeep Kaur

Academic Editor

PLOS One

Journal Requirements:

When submitting your revision, we need you to address these additional requirements.

1.Please ensure that your manuscript meets PLOS ONE's style requirements, including those for file naming. The PLOS ONE style templates can be found at

https://journals.plos.org/plosone/s/file?id=wjVg/PLOSOne_formatting_sample_main_body.pdf and

https://journals.plos.org/plosone/s/file?id=ba62/PLOSOne_formatting_sample_title_authors_affiliations.pdf

2. Please include captions for your Supporting Information files at the end of your manuscript, and update any in-text citations to match accordingly. Please see our Supporting Information guidelines for more information: http://journals.plos.org/plosone/s/supporting-information.

3. If the reviewer comments include a recommendation to cite specific previously published works, please review and evaluate these publications to determine whether they are relevant and should be cited. There is no requirement to cite these works unless the editor has indicated otherwise.

Additional Editor Comments:

Thank you for submitting this protocol addressing cultural awareness and cultural safety in Canadian speech-language pathology practice. The topic is timely, relevant, and has the potential to contribute meaningfully to the literature on culturally responsive healthcare and professional practice. The proposed scoping review addresses an important gap and is grounded in an appropriate methodological framework.

However, several aspects of the protocol require further clarification and methodological detail before the manuscript can be considered for publication.

1. Population–Concept–Context (PCC) Framework

The protocol would benefit from a clearer presentation of the Population, Concept, and Context (PCC) framework that underpins the review question. While these elements can be inferred from the text, explicitly presenting them in a dedicated table would improve transparency, methodological rigor, and alignment with established scoping review guidance. The PCC framework should clearly identify the target population, the concepts being examined, and the contextual boundaries of the review.

2. Stakeholder Consultation

The manuscript indicates stakeholder involvement; however, important details regarding this process are currently lacking. The authors should clearly specify:

* Who the stakeholders are.

* The rationale for selecting these stakeholders.

* At what stage(s) of the review process consultation will occur.

* The objectives of stakeholder consultation.

* How stakeholder feedback will be documented, analyzed, and incorporated into the review findings.

Providing this information is important for transparency and reproducibility.

3. Grey Literature Search Strategy

The protocol currently provides insufficient detail regarding the planned grey literature search. The authors should describe:

* Sources that will be searched (e.g., professional associations, government reports, institutional repositories, policy documents, dissertations, conference proceedings).

* Search procedures and search terms.

* Inclusion and exclusion criteria for grey literature.

* Methods for documenting and reporting grey literature retrieval.

Without these details, the review may be difficult to replicate.

4. Language Restrictions

The review focuses on the Canadian context, where both English and French are official languages. The authors should either:

* Expand the search strategy to include French-language publications, or

* Provide a clear methodological justification for restricting the review to English-language sources and discuss the potential implications of this decision.

Failure to address this issue may result in the omission of relevant evidence.

5. Publication Date Range

There appears to be inconsistency in the stated date limits of the search strategy. Different sections of the manuscript refer to different publication periods (2000–2025 and 2000–2026). The authors should ensure consistency throughout the manuscript and clearly justify the selected timeframe.

6. Data Management and Availability

As this is a protocol paper, the manuscript should provide greater clarity regarding management and sharing of the data generated during the review process. The authors should specify:

* What review data will be generated.

* Where extracted data, screening records, and review materials will be stored.

* Whether these materials will be publicly available upon completion of the review.

* How the protocol complies with PLOS ONE data-sharing requirements.

Addressing these points will improve transparency and reproducibility.

Overall, the study addresses an important topic and the proposed review is feasible and potentially valuable. However, additional methodological detail and clarification are necessary to ensure transparency, reproducibility, and adherence to current scoping review standards.

[Note: HTML markup is below. Please do not edit.]

Reviewers' comments:

Reviewer's Responses to Questions

**Comments to the Author**

1. Does the manuscript provide a valid rationale for the proposed study, with clearly identified and justified research questions?

The research question outlined is expected to address a valid academic problem or topic and contribute to the base of knowledge in the field.

Reviewer #1: Yes

**********

2. Is the protocol technically sound and planned in a manner that will lead to a meaningful outcome and allow testing the stated hypotheses?

The manuscript should describe the methods in sufficient detail to prevent undisclosed flexibility in the experimental procedure or analysis pipeline, including sufficient outcome-neutral conditions (e.g. necessary controls, absence of floor or ceiling effects) to test the proposed hypotheses and a statistical power analysis where applicable. As there may be aspects of the methodology and analysis which can only be refined once the work is undertaken, authors should outline potential assumptions and explicitly describe what aspects of the proposed analyses, if any, are exploratory.

Reviewer #1: Yes

**********

3. Is the methodology feasible and described in sufficient detail to allow the work to be replicable?

Descriptions of methods and materials in the protocol should be reported in sufficient detail for another researcher to reproduce all experiments and analyses. The protocol should describe the appropriate controls, sample size calculations, and replication needed to ensure that the data are robust and reproducible.

Reviewer #1: Yes

**********

4. Have the authors described where all data underlying the findings will be made available when the study is complete?

The PLOS Data policy requires authors to make all data underlying the findings described in their manuscript fully available without restriction, with rare exception, at the time of publication. The data should be provided as part of the manuscript or its supporting information, or deposited to a public repository. For example, in addition to summary statistics, the data points behind means, medians and variance measures should be available. If there are restrictions on publicly sharing data—e.g. participant privacy or use of data from a third party—those must be specified.

Reviewer #1: No

**********

5. Is the manuscript presented in an intelligible fashion and written in standard English?

PLOS ONE does not copyedit accepted manuscripts, so the language in submitted articles must be clear, correct, and unambiguous. Any typographical or grammatical errors should be corrected at revision, so please note any specific errors here.

Reviewer #1: Yes

**********

6. Review Comments to the Author

Please use the space provided to explain your answers to the questions above and, if applicable, provide comments about issues authors must address before this protocol can be accepted for publication. You may also include additional comments for the author, including concerns about research or publication ethics.

You may also provide optional suggestions and comments to authors that they might find helpful in planning their study.

(Please upload your review as an attachment if it exceeds 20,000 characters)

Reviewer #1: The manuscript is a great effort to know the cultural awareness among practicing SLPs in Canada. Though the methodology is strong, there are a few things that caught my attention:

1. The authors can introduce a Population, Concept and Context (PCC) table in the framework to improve methodological clarity.

2. The authors do not specify to who the stakeholders are, the purpose of their involvement, when they will be consulted during the review process, or how their feedback will be incorporated into the study.

3. The authors have not specified on the methodology employed for conducting grey literature search.

4. As French is one of the official languages of Canada, the authors may consider expanding the search to include studies published in French or provide a rationale for restricting the review to English-language

5. There appears to be an inconsistency in the publication date range, at one place it reads 2000-2025 and in the other it is mentioned as 2000-2026. The authors need to clarify the same.

**********

7. PLOS authors have the option to publish the peer review history of their article (what does this mean?). If published, this will include your full peer review and any attached files.

If you choose “no”, your identity will remain anonymous but your review may still be made public.

**Do you want your identity to be public for this peer review?**  For information about this choice, including consent withdrawal, please see our Privacy Policy.

Reviewer #1: **Yes:**  Prashasti P. Poovaiah

**********

[NOTE: If reviewer comments were submitted as an attachment file, they will be attached to this email and accessible via the submission site. Please log into your account, locate the manuscript record, and check for the action link "View Attachments". If this link does not appear, there are no attachment files.]

To ensure your figures meet our technical requirements, please review our figure guidelines: https://journals.plos.org/plosone/s/figures

You may also use PLOS’s free figure tool, NAAS, to help you prepare publication quality figures: https://journals.plos.org/plosone/s/figures#loc-tools-for-figure-preparation.

NAAS will assess whether your figures meet our technical requirements by comparing each figure against our figure specifications.

# Author comment: Author response to Decision Letter 1

**DOI:** 10.1371/journal.pone.0347949.r002

16 Jun 2026

Response to Reviewer and Editor

We would like to thank the Academic Editor and Reviewers for their time and thoughtful feedback on our manuscript. We appreciate the constructive comments and suggestions provided, which have helped strengthen the clarity, rigor, and transparency of the protocol.

We have carefully reviewed all comments and have revised the manuscript accordingly. All changes have been incorporated into the revised manuscript and are detailed below.

1. Population–Concept–Context (PCC) Framework

The protocol would benefit from a clearer presentation of the Population, Concept, and Context (PCC) framework that underpins the review question. While these elements can be inferred from the text, explicitly presenting them in a dedicated table would improve transparency, methodological rigor, and alignment with established scoping review guidance. The PCC framework should clearly identify the target population, the concepts being examined, and the contextual boundaries of the review.

Response: The table below has been added to the manuscript and labelled as Table 1

Component Description

Population Indigenous Peoples (First Nations, Inuit, Métis) and other equity deserving populations, including racialized groups, immigrants and refugees, 2SLGBTQIA+ individuals, and other marginalized communities experiencing structural or systemic inequities in Canada.

Concept Culturally responsive, culturally safe, trauma informed, and equity oriented practices, frameworks, assessments, and interventions in speech language pathology. This includes concepts across the cultural continuum such as cultural awareness, sensitivity, competence, humility, responsiveness, and safety.

Context Speech language pathology practice in Canada, including services delivered across healthcare, educational, community, and private practice settings, across the lifespan.

2. Stakeholder Consultation

The manuscript indicates stakeholder involvement; however, important details regarding this process are currently lacking. The authors should clearly specify:

Response: Thank you for this helpful suggestion. We have revised the manuscript to explicitly address each component of stakeholder consultation as outlined below:

• Who the stakeholders are: We now specify that stakeholders may include community partners and knowledge users such as community members, Elders, and individuals connected to Indigenous communities, newcomer and immigrant populations, and other equity deserving groups.

• Rationale for selecting these stakeholders: The rationale is to support culturally appropriate and respectful representation of findings, ensuring that the language, framing, and presentation of results align with principles of cultural safety and do not misrepresent the communities discussed.

• Stage(s) of consultation: Consultation will occur during the write up stages of the review.

• Objectives of stakeholder consultation: The objectives are to support culturally safe language use, ensure appropriate representation of communities, and identify any potentially unclear or inappropriate framing in how findings are presented.

• Documentation and incorporation of feedback: Stakeholder input will be documented in a manner that respects participant preferences. Feedback will inform revisions to language and presentation of findings but will not influence study selection, data extraction, or interpretation of results. Contributions will be acknowledged based on participant consent (named, collective acknowledgment, or not acknowledged).

We have also clarified that stakeholder consultation may not always be feasible, and the extent of consultation will be reported transparently with limitations noted in the manuscript.

3. Grey Literature Search Strategy

The protocol currently provides insufficient detail regarding the planned grey literature search.

Response:

Thank you for this valuable suggestion. We have revised the manuscript to expand the grey literature search strategy and explicitly address each component as outlined below:

• Sources to be searched: We now specify that grey literature will be identified through targeted searches of professional and regulatory organizations, government and health authority websites, community and non profit organizations, institutional repositories (e.g., ProQuest Dissertations and Theses), grey literature databases (e.g., Canada Commons), and Google searches.

• Search procedures and search terms: We clarify that combinations of keywords related to Speech Language Pathology, cultural responsiveness, and populations of interest will be used, and that search strings will be adapted for each source.

• Inclusion and exclusion criteria for grey literature: We specify that grey literature sources must meet the same Population–Concept–Context criteria as peer reviewed literature and provide substantive discussion of culturally responsive or culturally safe practices in Canadian S-LP practices and/or services.

• Documentation and reporting methods: We state that all grey literature searches will be documented, including search terms, dates, sources consulted, and records identified, and that these will be reported in the PRISMA flow diagram and described in the manuscript.

These revisions have been incorporated to improve transparency, rigor, and reproducibility.

4. Language Restrictions

The review focuses on the Canadian context, where both English and French are official languages. The authors should either:

* Expand the search strategy to include French-language publications, or

* Provide a clear methodological justification for restricting the review to English-language sources and discuss the potential implications of this decision.

Failure to address this issue may result in the omission of relevant evidence.

Response: Thank you for this important point. We have clarified our approach to language inclusion in the Methods section. Due to limitations in the research team’s ability to accurately interpret and analyze full-text French-language publications, the review has been restricted to English-language sources to ensure consistency and rigor in data extraction and analysis. We acknowledge that this decision may result in the exclusion of some relevant evidence, and we now explicitly identify this as a limitation of the review. To enhance transparency, relevant French-language records with available English abstracts, identified during screening will be documented and discussed in the final manuscript.

5. Publication Date Range

There appears to be inconsistency in the stated date limits of the search strategy. Different sections of the manuscript refer to different publication periods (2000–2025 and 2000–2026). The authors should ensure consistency throughout the manuscript and clearly justify the selected timeframe.

Response: Thank you for highlighting this inconsistency. We have revised the manuscript to ensure that the publication date range is reported consistently throughout. The selected timeframe (2000–2026) is now clearly and consistently specified across all sections of the manuscript.

6. Data Management and Availability

As this is a protocol paper, the manuscript should provide greater clarity regarding management and sharing of the data generated during the review process.

Response: Thank you for this important suggestion. We have added a dedicated Data Management and Availability section to the Methods to improve transparency and reproducibility. This section addresses each of the requested elements as follows:

• What review data will be generated: We specify that the review will generate data related to screening decisions (e.g., inclusion and exclusion records), extracted study characteristics, and synthesized outputs.

• Where data will be stored: We clarify that data will be managed using Covidence systematic review software and Microsoft Excel, and stored on secure, password-protected institutional systems.

• Public availability of materials: We state that relevant review data (e.g., extracted datasets and summary tables) will be made publicly available upon completion of the review where feasible, or provided upon reasonable request.

• Compliance with PLOS ONE data-sharing requirements: We note that no original data are generated at the protocol stage and confirm that the study complies with PLOS ONE data-sharing policies.

These revisions have been incorporated to enhance clarity, transparency, and reproducibility.

Overall, the study addresses an important topic and the proposed review is feasible and potentially valuable. However, additional methodological detail and clarification are necessary to ensure transparency, reproducibility, and adherence to current scoping review standards.

Attachment Submitted filename: Response to Reviewers .docx

# Editor report: Decision Letter 1

**DOI:** 10.1371/journal.pone.0347949.r003

**Authors:** Ramandeep Kaur

14 Jul 2026

From cultural awareness to safety in Canadian Speech‑Language Pathology practice: A Scoping Review Protocol

PONE-D-26-17409R1

Dear Dr. Komeili,

We’re pleased to inform you that your manuscript has been judged scientifically suitable for publication and will be formally accepted for publication once it meets all outstanding technical requirements.

Within one week, you’ll receive an e-mail detailing the required amendments. When these have been addressed, you’ll receive a formal acceptance letter and your manuscript will be scheduled for publication.

An invoice will be generated when your article is formally accepted. Please note, if your institution has a publishing partnership with PLOS and your article meets the relevant criteria, all or part of your publication costs will be covered. Please make sure your user information is up-to-date by logging into Editorial Manager at Editorial Manager® and clicking the ‘Update My Information' link at the top of the page. For questions related to billing, please contact billing support.

If your institution or institutions have a press office, please notify them about your upcoming paper to help maximize its impact. If they’ll be preparing press materials, please inform our press team as soon as possible -- no later than 48 hours after receiving the formal acceptance. Your manuscript will remain under strict press embargo until 2 pm Eastern Time on the date of publication. For more information, please contact onepress@plos.org.

Kind regards,

Ramandeep Kaur

Academic Editor

PLOS One

Additional Editor Comments (optional):

Reviewers' comments:

# Editor report: Acceptance letter

**DOI:** 10.1371/journal.pone.0347949.r004

**Authors:** Ramandeep Kaur

PONE-D-26-17409R1

PLOS One

Dear Dr. Komeili,

I'm pleased to inform you that your manuscript has been deemed suitable for publication in PLOS One. Congratulations! Your manuscript is now being handed over to our production team.

At this stage, our production department will prepare your paper for publication. This includes ensuring the following:

* All references, tables, and figures are properly cited

* All relevant supporting information is included in the manuscript submission,

* There are no issues that prevent the paper from being properly typeset

You will receive further instructions from the production team, including instructions on how to review your proof when it is ready. Please keep in mind that we are working through a large volume of accepted articles, so please give us a few days to review your paper and let you know the next and final steps.

Lastly, if your institution or institutions have a press office, please let them know about your upcoming paper now to help maximize its impact. If they'll be preparing press materials, please inform our press team within the next 48 hours. Your manuscript will remain under strict press embargo until 2 pm Eastern Time on the date of publication. For more information, please contact onepress@plos.org.

You will receive an invoice from PLOS for your publication fee after your manuscript has reached the completed accept phase. If you receive an email requesting payment before acceptance or for any other service, this may be a phishing scheme. Learn how to identify phishing emails and protect your accounts at https://explore.plos.org/phishing.

If we can help with anything else, please email us at customercare@plos.org.

Thank you for submitting your work to PLOS ONE and supporting open access.

Kind regards,

PLOS ONE Editorial Office Staff

on behalf of

Dr. Ramandeep Kaur

Academic Editor

PLOS One
